# Quality of reporting of robot-assisted cholecystectomy in relation to the IDEAL recommendations: systematic review

**DOI:** 10.1093/bjsopen/zrac116

**Published:** 2022-10-25

**Authors:** Emily N Kirkham, Conor S Jones, George Higginbotham, Sarah Biggs, Ffion Dewi, Lauren Dixon, Marc Huttman, Barry G Main, Jozel Ramirez, Harry Robertson, Darren L Scroggie, Benjamin Zucker, Jane M Blazeby, Natalie S Blencowe, Samir Pathak, RoboSurg Collaborative, A Vallance, A Wilkinson, A Smith, A Torkington, A Jones, A Abbas, B G Main, B Zucker, B Turner, C S Jones, C Thomas, C Hoffmann, D L Scroggie, D Henshall, E N Kirkham, E Boden, E Gull, E Sewart, F Dewi, F Wood, F Loro, F Hollowood, G Fowler, G Higginbotham, G Sellers, H Robertson, H Richards, I Hughes, I Handa, J M Blazeby, J Olivier, J Ramirez, J Rees, K Chalmers, K Siang Lee, L Dixon, L Leandro, L Paynter, L Huppler, L Gourbault, M Huttman, M Wijeyaratne, M Dewhurst, M Shah, M Kiandee, M Dada, N S Blencowe, O Brewster, P Lok, R Winayak, R Ranat, R Macefield, R Purves, R Lawrence, R Millar, S Biggs, S Lawday, S Dalmia, S Cousins, S Pathak, S Rozwadowski, T Robinson, T Perra, T Wei Leow, T Brankin-Frisby, W Baker, W Hurst, Y Embury Young

**Affiliations:** Bristol Centre for Surgical Research, Population Health Sciences, University of Bristol, Bristol, UK; Musgrove Park Hospital, Taunton, UK; Bristol Centre for Surgical Research, Population Health Sciences, University of Bristol, Bristol, UK; North Bristol NHS Foundation Trust, Bristol, UK; North Bristol NHS Foundation Trust, Bristol, UK; University Hospitals Bristol and Weston NHS Foundation Trust, Bristol, UK; University Hospitals Bristol and Weston NHS Foundation Trust, Bristol, UK; Bristol Centre for Surgical Research, Population Health Sciences, University of Bristol, Bristol, UK; University Hospitals Bristol and Weston NHS Foundation Trust, Bristol, UK; Bristol Centre for Surgical Research, Population Health Sciences, University of Bristol, Bristol, UK; University College Hospital, University College London Hospitals NHS Foundation Trust, London, UK; Bristol Centre for Surgical Research, Population Health Sciences, University of Bristol, Bristol, UK; University Hospitals Bristol and Weston NHS Foundation Trust, Bristol, UK; Bristol Dental School, University of Bristol, Bristol, UK; NIHR Bristol Biomedical research centre, Bristol, UK; Bristol Centre for Surgical Research, Population Health Sciences, University of Bristol, Bristol, UK; University Hospitals Bristol and Weston NHS Foundation Trust, Bristol, UK; Bristol Centre for Surgical Research, Population Health Sciences, University of Bristol, Bristol, UK; Imperial College Healthcare NHS Trust, London; Bristol Centre for Surgical Research, Population Health Sciences, University of Bristol, Bristol, UK; University Hospitals Bristol and Weston NHS Foundation Trust, Bristol, UK; Bristol Centre for Surgical Research, Population Health Sciences, University of Bristol, Bristol, UK; University Hospitals Bristol and Weston NHS Foundation Trust, Bristol, UK; Bristol Centre for Surgical Research, Population Health Sciences, University of Bristol, Bristol, UK; NIHR Bristol Biomedical research centre, Bristol, UK; Bristol Centre for Surgical Research, Population Health Sciences, University of Bristol, Bristol, UK; University Hospitals Bristol and Weston NHS Foundation Trust, Bristol, UK; NIHR Bristol Biomedical research centre, Bristol, UK; St James’s University Hospital, Leeds Teaching Hospitals NHS Trust, Leeds, UK

## Abstract

**Introduction:**

Robotic cholecystectomy (RC) is a recent innovation in minimally invasive gallbladder surgery. The IDEAL (idea, development, exploration, assessment, long-term study) framework aims to provide a safe method for evaluating innovative procedures. This study aimed to understand how RC was introduced, in accordance with IDEAL guidelines.

**Methods:**

Systematic searches were used to identify studies reporting RC. Eligible studies were classified according to IDEAL stage and data were collected on general study characteristics, patient selection, governance procedures, surgeon/centre expertise, and outcome reporting.

**Results:**

Of 1425 abstracts screened, 90 studies were included (5 case reports, 38 case series, 44 non-randomized comparative studies, and 3 randomized clinical trials). Sixty-four were single-centre and 15 were prospective. No authors described their work in the context of IDEAL. One study was classified as IDEAL stage 1, 43 as IDEAL 2a, 43 as IDEAL 2b, and three as IDEAL 3. Sixty-four and 51 provided inclusion and exclusion criteria respectively. Ethical approval was reported in 51 and conflicts of interest in 34. Only 21 reported provision of training for surgeons in RC. A total of 864 outcomes were reported; 198 were used in only one study. Only 30 reported a follow-up interval which, in 13, was 1 month or less.

**Conclusion:**

The IDEAL framework was not followed during the adoption of RC. Few studies were conducted within a research setting, many were retrospective, and outcomes were heterogeneous. There is a need to implement appropriate tools to facilitate the incremental evaluation and reporting of surgical innovation.

## Teaser

Robotic cholecystectomy (RC) is a recent innovation in minimally invasive gallbladder surgery. Disparity between adoption of new techniques and robust evaluation has been observed in other areas of surgery and we sought to evaluate whether IDEAL guidelines were followed in the context of RC. We found that the IDEAL framework was not followed, few studies were conducted within a research setting, many were retrospective, and outcomes were heterogeneous. This lack of incremental evidence impairs surgeons’ ability to draw meaningful conclusions, undermining shared decision-making with patients. There is a need to implement appropriate tools to facilitate the incremental evaluation and reporting of surgical innovation

## Introduction

Approximately 70 000 cholecystectomies are undertaken each year in England at a cost of around £111 million^1^. More than 90 per cent of these are performed using laparoscopic techniques^1^. Laparoscopic cholecystectomy (LC) typically results in less postoperative pain, faster recovery, improved cosmesis, and a shorter hospital stay compared with open surgery^2^. Single-incision laparoscopic cholecystectomy (SILC) was developed in 2010^3^ in an attempt to further improve cosmesis and decrease postoperative pain^4^; however, ergonomic limitations and a lack of clear clinical benefit have hindered its adoption into routine practice^5–7^.

Robotic cholecystectomy (RC) is the most recent technological innovation for minimally invasive gallbladder surgery. It is performed through single or multiple small incisions, by an operating surgeon seated at a console away from the sterile field^8^. RC has perceived benefits, including enhanced tactile feedback, reduced musculoskeletal strain on the surgeon, better exposure, easier manipulation of the instruments, high-definition three-dimensional visualization, and fewer instrument collisions^5,8^. Due to these purported advantages, it is becoming increasingly popular; in the USA, rates of RC increased from 0.02 per cent of all cholecystectomies performed in 2008 to 3.2 per cent in 2017^9^. This increase may in part be due to surgeons using RC as a means of developing their robotic skills for more complex operations^10^; however, convincing evidence of clinical benefit over conventional laparoscopic methods has not been forthcoming^11,12^. The disparity between adoption of new techniques and robust evaluation has been observed in other areas of gastrointestinal surgery^13^, leading to calls for tighter regulation of the field^14^. Although surgical robots are considered devices and subject to regulatory approval, there is currently no requirement for individual procedures such as RC to undergo robust clinical evaluation before implementation in clinical practice.

The idea, development, exploration, assessment, long-term follow-up (IDEAL) framework was developed in 2009 and updated in 2019^15^, to provide a stepwise approach for the evaluation and reporting of innovative surgical procedures (*Table 1*).

**Table 1:** Summary of idea, development, exploration, assessment, long-term study stages and recommendations

	IDEA (1)	Development (2a)	Evaluation (2b)	Assessment (3)	Long-term (4)
Purpose	Proof of concept	Establish technical details and replicate early results	Learning	Assessment	Surveillance
Design	Structured case report	Prospective case series	Prospective comparative case series, feasibility RCT	RCT	Audit, registry, database
Number of patients	1	Few (<30)	Many (>30)	Guided by sample size calculation	Often large numbers
Inclusion criteria	Highly selected	Selected	Widening	Wide	Wide
Technical modifications	Report success and failures (inception)	Modifications allowed (development)	Modifications allowed (refinement)	No further modifications (stable)	No further modifications (stable)
Considered innovative procedure	Yes*	Yes*	Yes*	No	No
Surgeon and centre expertise	Details of pre-human work	Details of surgeon training	Details of mentoring and learning curve	Surgeons should be past learning curve	Surgeons should be past learning curve
Outcomes	Proof of concept; technical achievement; dramatic success; adverse events, surgeon views of the procedure	Mainly safety; technical and procedural success	Safety; clinical outcomes (specific/graded); short-term outcomes; patient-centred/reported outcomes; feasibility outcomes	Clinical outcomes (specific and graded); potential patient-reported outcomes, health economic outcomes	Rare events; long-term outcomes; quality assurance

Adapted from Hirst 2019^15^ and Currie 2015^16^.

* Specific consent regarding innovation is required. RCT, randomized clinical trial.

Specific recommendations include details about patient selection, governance measures, surgeon expertise, and standardized outcome reporting, all which are critical to the safe introduction of new surgical procedures. By providing a stepwise framework to report the evolution of innovations, IDEAL seeks to facilitate incremental learning^17^, whereby researchers build on previous reports and add value to the existing evidence base. It is presently unclear whether this process occurred during the adoption and evaluation of RC.

The aims of this study are to understand how RC has been adopted into clinical practice, and to establish whether the evaluation and reporting of RC occurred in accordance with IDEAL guidelines.

## Methods

The methods are based on a previously published protocol that aimed to investigate the introduction of a robotic procedure for diseases of the oropharynx^5^. Reporting was conducted in line with PRISMA 2020 guidelines^18^ (*Tables S1* and *S2*).

### Search strategy and study selection

Searches were undertaken in MEDLINE, Embase, Cochrane Library, and Web of Science databases, from inception to February 2020. Searches consisted of subject headings and text words, combining terms for ‘robotic surgery’ with ‘cholecystectomy’ using the Boolean operator ‘AND’ (*Table S3*).

### Study eligibility

Searches were limited to studies of adults aged 18 years or older and written in English. All primary research study designs (such as case reports, case series, and comparative studies) were eligible for inclusion. Presentations and conference abstracts were excluded because of the high probability of incomplete data. Further exclusions included studies where the main focus was not the surgical procedure (such as anaesthesia, perioperative physiotherapy, or nutrition); describing indications for cholecystectomy other than cholelithiasis or polyps (such as cancer); where a combination of robotic procedures was described (such as when results of RC were reported alongside other robotic procedures and could not be separated); and investigating robotic camera holders rather than RC itself.

### Identification and selection of papers

Search results were de-duplicated and uploaded to Rayyan software (Rayyan - a web and mobile app for systematic reviews)^19^. Titles and abstracts were screened independently by at least two authors. The full-text versions of papers retained after title and abstract screening were further assessed for eligibility. Disagreements were first discussed between the reviewers, and any unresolved conflicts referred to the senior authors (N.B. and S.P.); the final decision was the majority opinion. Data from full-text papers were extracted independently by at least two assessors.

### Data collection

Data collection was based on IDEAL recommendations and included information about general study characteristics, patient selection, regulatory and governance arrangements, centre and operator expertise, and outcome reporting^13,20^.

#### General study characteristics and identification of IDEAL stage

The study design, year, and journal of publication, country of origin, and number of participating centres and patients were extracted. The presence and nature of comparison interventions and the type of robotic device used in each study were documented.

Where authors reported an IDEAL stage, it was recorded. Where this information was not provided, a flow diagram designed by the IDEAL Collaboration was used to establish the IDEAL stage^21^. Any difficulties assigning IDEAL stages to papers were recorded. Risk of bias assessments were undertaken for randomized clinical trials (RCTs) using the revised Cochrane Risk of Bias tool^22^.

Any reported rationale for why the study was undertaken was documented in the following categories: assessment of safety and efficacy; support for regulatory approval (such as the Medicines and Healthcare Products Regulatory Agency); description of technique; evaluation of learning curves; description of a centre’s experience; prediction of patient outcomes; and/or ‘other’.

#### Patient selection

Inclusion and exclusion criteria for patients undergoing RC were documented for each study. The number of patients declining RC was recorded, along with any stated reasons.

#### Regulatory and governance arrangements

The reporting of conflicts of interest, study funding and governance approvals (such as ethics committees, institutional review boards, or clinical effectiveness committees) was collected. Statements relating to patient consent, and whether patients were specifically informed of the innovative nature of RC, or of modifications made to the surgical technique, were recorded.

#### Centre and surgeon expertise

Information about centre expertise, such as the volume of robotic and non-robotic cholecystectomies undertaken at the institution(s), was recorded. Information about the number of surgeons performing the operation, and the expertise of those surgeons was also extracted, including their grade and experience with RC, and any details of specific training and mentorship in RC.

#### Outcome selection, measurement, and reporting

Outcomes reported in each manuscript were recorded verbatim and categorized into domains by two researchers (E.K. and C.S.J.; *Table S4*). To determine the number of distinct outcomes, those with the same meaning but different wording, were rationalized within each domain. Where reported, the duration of follow-up for each study was documented.

### Data synthesis

Results were summarized in a narrative synthesis, with descriptive statistics where appropriate. The study did not aim to investigate the effectiveness of RC, therefore meta-analyses were not performed. To evaluate whether studies’ rationale and outcomes evolved over time, data were presented by IDEAL stage.

## Results

**Fig. 1:**
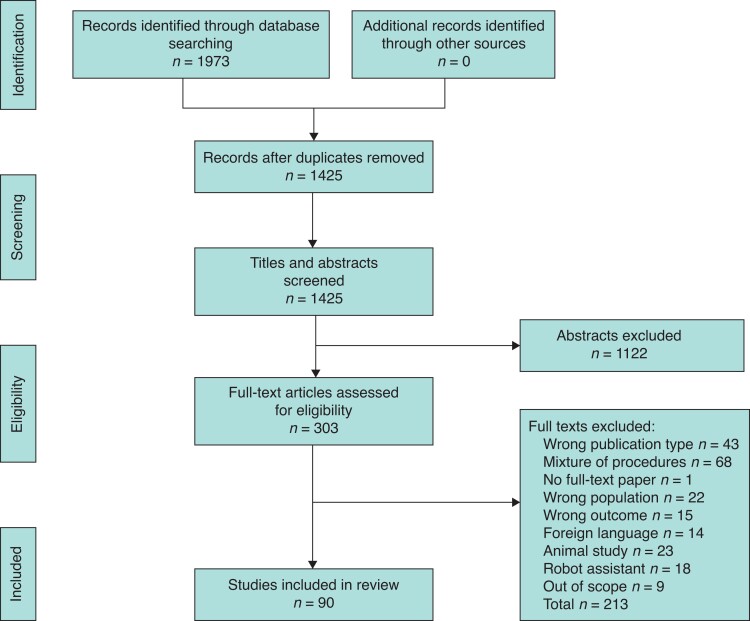
PRISMA diagram

Of 1425 abstracts and 303 full-text articles screened, a total of 90 articles, published between 2001 and 2020, were included (*Fig. 1*). There were two large database studies, collectively reporting short-term outcomes from 827 386 patients (823 807 LC and 3579 RC). The remaining 88 studies included a total of 15 074 patients (median 58, range 1–3255), of which 7009 underwent RC (median 38, range 1–925) and 7867 LC (median 50, range 5–3149).

### General study characteristics

Among the 90 studies there were five case studies, 38 case series, 44 non-randomized comparative studies and three RCTs. Most studies were single-centre (*n* = 64) and only 15 were prospective (*Table 2*). All three RCTs compared RC with LC, and were published in 2014^23^ (single-centre, *n* = 22), 2015^24^ (single-centre, *n* = 60), and 2017^25^ (multicentre, *n* = 136; *Table S5*). The risk of bias was unclear in two^23,25^, and in one^24^ there was a large (more than 20 per cent) loss to follow-up.

**Table 2:** Characteristics of included studies

	Number of studies (*n* = 90)
**Non-comparative studies**	** **
Case reports	5
Case series	38
Prospective	12
Retrospective	15
Mixed	2
Not specified	9
**Comparative studies**	** **
Non-randomized comparative studies	44
Prospective	3
Retrospective	34
Mixed	3
Not specified	4
Randomized clinical trials	3
**Number of centres**	** **
Single	64
Multiple	6
Not specified	20
**Type of centres**	** **
Tertiary/specialist	28
Secondary/general	1
Mixed	1
Not specified	59
**Country of study**	** **
USA	40
South Korea	10
Switzerland	9
The Netherlands	8
Italy	6
Austria	3
Turkey	2
Germany	2
Taiwan	2
Other*	7
**Comparator interventions**	** **
Laparoscopic cholecystectomy	31
Single-incision cholecystectomy	11
RC	3
Single *versus* multiple port	1
With and without cholangiography	1
Emergency *versus* elective	1

* Brazil, Hong Kong, France, UK, Canada, Greece, Multiple: all *n* = 1. RC, robotic cholecystectomy.

The most commonly used robots were Da Vinci systems (Intuitive Surgical (California, US)., 66). Seventeen studies provided no description of the system used.

**Fig. 2:**
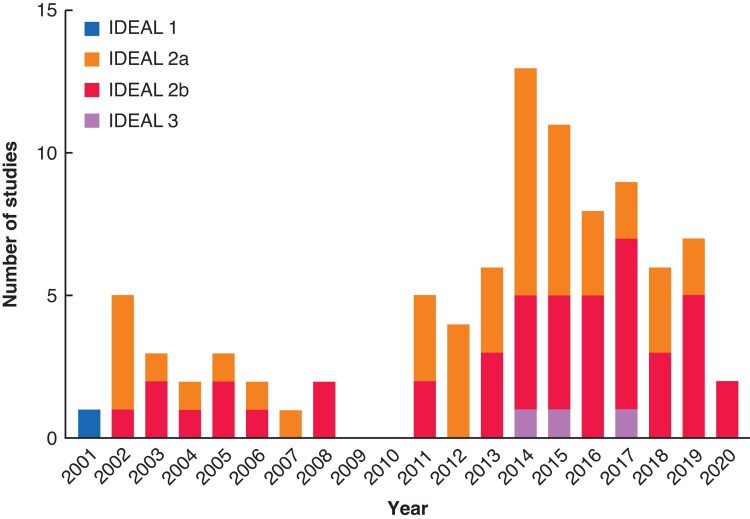
Progression of idea, development, exploration, assessment, long-term study stage of included studies over time

No studies reported an IDEAL stage. The first study (a case series of 20 patients published in 2001) was considered to be IDEAL stage 1. Forty-three studies were identified as IDEAL 2a, 43 as IDEAL 2b, and three as IDEAL 3 (the RCTs), with no studies meeting the criteria for IDEAL stage 4. We experienced difficulties assigning IDEAL stages to many of the included papers. Overall, 49 studies were retrospective in nature and therefore did not strictly meet the IDEAL criteria, and had a further problem was the lack of detail about technique description or modifications, making it difficult to differentiate between stage 2a and 2b. Although two studies undertook data analysis from large databases, they only included information about short-term adverse events and, as such, did not meet the criteria for IDEAL stage 4. Although the number of IDEAL 2b studies has increased over time, only three were conducted prospectively. IDEAL stage 2a studies are still being conducted, despite the fact that the first RCT was published in 2014. There is, therefore, minimal evidence of evolution of study design as per the IDEAL recommendations (*Fig. 2*).

Of the 90 studies, 73 reported a rationale. Most commonly, this was to assess safety, efficacy, and adverse events (*n* = 38). Others included descriptions of a centre’s experience (*n* = 18), prediction of outcomes (*n* = 13), evaluation of the learning curve (*n* = 11), and/or descriptions of the surgical technique (*n* = 7). There was no correlation between study rationale and IDEAL stage (the rationale did not evolve despite advancing IDEAL stage; *Table 3*).

**Table 3:** Reporting of study rationale by idea, development, exploration, assessment, long-term study stage

Rationale	IDEAL 1 (*n* = 1)	IDEAL 2a (*n* = 43)	IDEAL 2b (*n* = 43)	IDEAL 3 (*n* = 3)
**Studies reporting a rationale**	1 (100)	33 (77)	37 (86)	3 (100)
Rationale(s)* of study				
Safety and efficacy	1	15	21	3
Technique description	1	4	2	0
Evaluation of learning curve	0	5	5	0
Description of centre's experience	0	9	9	0
Predicting patient outcomes	0	6	7	0

values are *n* (%). *Some studies reported multiple rationales. IDEAL (idea, development, exploration, assessment, long-term study) suggest that each IDEAL stage should fulfil the following purposes: IDEAL 1, proof of concept; IDEAL 2a, establish technical details and replicate early results; IDEAL 2b, learning; IDEAL 3, assessment; IDEAL 4, surveillance (*Table 1*).

### Patient selection

Sixty-four and 51 studies provided inclusion and exclusion criteria respectively (*Tables 4* and *5*). Eight studies reported that there were no exclusion criteria. A total of 15 studies described how patients were selected for robotic surgery over conventional approaches: availability of the robot (*n* = 8), surgeon’s discretion (*n* = 4), willingness to pay (*n* = 1), the time interval of recruitment (before and after the robot became available, *n* = 1), and one study stated that there were no formal selection criteria. No studies specifically commented on the number of patients declining RC.

**Table 4:** Summary of inclusion criteria reported in the included studies

Inclusion criteria	Number of studies (*n* = 64)
**Disease-related**	
Indication	46
Gallstones	38
Other benign non-inflammatory diseases (biliary dyskinesia, adenomyomatosis, polyps)	14
Acute cholecystitis	9
Chronic cholecystitis	5
Gallstone pancreatitis	5
Choledocholithiasis	1
‘Gallbladder disease’ (unspecified)	6
Symptoms	31
Symptomatic	30
Asymptomatic	1
**Patient-related**	
Age (years)	20
>18	10
18–80	9
23–78	1
BMI	6
<30	2
>30	1
>25	1
No limit	2
Co-morbidity	9
ASA grade 1–3	5
ASA grade 1–2	2
Mild-moderate severity of illness score	1
Based on anaesthetic risk	1
No previous upper abdominal surgery	3
**Surgery-related**	
Urgency	16
Elective	15
Emergency and elective	1
Fluorescent imaging	2
**Other**	
Consent	7
Financial	1

**Table 5:** Summary of exclusion criteria reported in the included studies

	Number of studies (*n* = 51)
**Disease-related**	
Indication	51
Acute cholecystitis	24
CBD stone(s)	12
Pancreatitis	10
Gallbladder malignancy	6
Gallbladder empyema	1
Acute systemic illness	6
Deranged liver function test	3
**Patient-related**	
Age (years)	3
<18	3
Co-morbidity	16
Cognitive impairment	7
High anaesthetic risk	7
Liver cirrhosis	6
Coagulopathy	5
ASA grade >2	3
ASA grade 4	1
Other	3
Previous upper abdominal surgery	22
BMI	6
Not a cause for exclusion	2
Severe/morbid obesity	2
>32	1
>33	1
Pregnancy	12
Iodine allergy	4
Ability to consent	3
**Surgery-related**	
Urgency	3
Emergency surgery	3
Co-intervention	6
Concurrent surgery	5
Intraoperative cholangiogram	1
Adverse event	3
Conversion to open	2
Anaesthetic complication	1
Single incision	1
**Other**	
‘No exclusion criteria’	8
Consent	3
Incomplete records	3

### Regulatory and governance arrangements

Ethical approval was reported in 51 of the 90 studies (institutional review boards, *n* = 41 and ethics committee, *n* = 10) and four reported registration within a trials register (ClinicalTrials.gov, *n* = 3 and Australian New Zealand Clinical Trials Registry, *n* = 1). Conflicts of interest were common, with 11 studies funded by the robot manufacturer and a further 23 reporting conflicts of interest between the author(s) and the manufacturer.

Although patient consent for study participation was explicitly documented in 42 studies, just four stated that patients were informed of the innovative nature of RC^10,26–28^. Of the 10 studies reporting modifications to the robotic technique during the study, none reported that patients were informed of this.

### Centre and operator expertise

Four studies defined the participating centres’ usual caseload for RC (range 50–500 per year). The number of surgeons performing robotic surgery was reported in 51 studies (median 2, range 1–42). The grade of operating surgeon(s) was reported in 12 studies (consultant/attending, *n* = 2 and mixed trainee and consultant, *n* = 10). Provision of training in RC was reported in 21 studies, mostly consisting of animal-based (*n* = 12), simulation (*n* = 10), and dry laboratory (*n* = 6; *Table 6*). Proctorship and dual-consultant operating were each reported in four studies.

**Table 6:** Details of training in robotic cholecystectomy reported within included studies

Author, year*	Pre-clinical				Clinical			
	Simulation	Dry laboratory	Cadaver	Animal	Observation of surgical cases	Assisting	Proctorship	Other
Kim, 2002^29^				✓				✓
Bodner, 2002^30^		✓						
Ruurda, 2002^31^	✓							✓
Hourmont, 2003^32^	✓				✓		✓	✓
Vuilleumier, 2003^33^			✓	✓				
Miller, 2004^34^	✓							
Nio, 2004^35^		✓						
Caratozzolo, 2005^36^				✓				
Vidovszky, 2006^37^		✓				✓		
Breitenstein, 2008^38^	✓			✓				
Spinoglio, 2012^39^	✓			✓				
Pietrabissa, 2012^26^				✓				
Angus, 2014^40^	✓	✓		✓	✓		✓	✓
Nelson, 2014^41^		✓			✓	✓		
Ayloo, 2014^42^		✓		✓				
Juza, 2014^43^	✓			✓		✓		✓
Gonzalez, 2016^44^			✓	✓			✓	
Ayabe, 2018^45^	✓					✓		✓
O'Leary, 2018^46^	✓				✓		✓	✓
Melling, 2019^47^	✓			✓				
Lee, 2019^48^			✓	✓				
Total (*n* = 21)	10	6	3	12	4	4	4	8

* Only studies that reported any training information are included.

### Outcome selection, measurement, and reporting

A total of 842 outcomes were reported across all included studies. Of these, there were 280 distinct outcomes, of which 198 were used in only one study each. No single outcome, or outcome domain, was reported in all studies (median 8, interquartile range 6–12; *Table 7*). Outcomes relating to technical/operative factors (*n* = 377, 87 studies), complications (*n* = 236, 80 studies), and health economics (*n* = 139, 70 studies) were used most frequently. Four studies reported surgeon-focused outcomes and 24 provided patient-centred outcomes. There was no clear progression in the type of outcomes reported with advancing IDEAL stage (*Table 7*). Only 30 studies reported a follow-up interval (range 14 days to 46 months) and of these, 13 lasted 1 month or less.

**Table 7:** The selection and reporting of outcomes by domain and idea, development, exploration, assessment, long-term study stage

Domain	IDEAL 1 (*n* = 1)		IDEAL 2a (*n* = 43)		IDEAL 2b (*n* = 43)		IDEAL 3 (*n* = 3)		Total
	Outcomes	Studies	Outcomes	Studies	Outcomes	Studies	Outcomes	Studies	Outcomes
Complications	1	1	105	42	125	35	5	2	236
Technical	4	1	211	42	158	42	4	2	377
Health economic	0	0	33	29	105	40	1	1	139
Patient-centred	0	0	18	12	17	10	6	2	41
Laboratory and imaging	0	0	4	3	7	4	5	1	16
Surgeon-centred	0	0	13	3	1	1	0	0	14
Survival	0	0	5	5	6	6	0	0	11
Trends and learning curve	0	0	2	2	2	2	0	0	4
Pathological	0	0	3	3	1	1	0	0	4
Total	5	-	394	-	422	-	21	-	842

IDEAL (idea, development, exploration, assessment, long-term study) suggest the following outcomes at each stage: IDEAL 1, proof of concept, technical achievement, dramatic success, adverse events, surgeon views of the procedure; IDEAL 2a, mainly safety, technical and procedural success; IDEAL 2b, safety, clinical outcomes (specific/graded), short-term outcomes, patient-centred/reported outcomes, feasibility outcomes; IDEAL 3, clinical outcomes (specific and graded), potential patient-reported outcomes, health economic outcomes (*Table 1*).

## Discussion

This comprehensive review of the reporting of the adoption of RC summarizes information from 90 studies published between 2001 and 2020. The current evidence base for RC is formed largely by retrospective observational studies from single centres. Although three RCTs were identified, they were small and poorly designed. Most studies aimed to assess the safety of RC, with little evolution of study rationale or design that would be expected based on synthesis of preceding evidence. Details of regulatory and governance arrangements were infrequently reported, and conflicts of interest were common. Selection criteria were inconsistently reported, limiting understanding of which patients were offered the new procedure and why. Provision of training in RC was poorly reported with only four studies reporting any ongoing monitoring or proctorship. Outcome selection and reporting was heterogeneous, with 198 of the outcomes used just once. This review highlights that RC has been adopted into clinical practice without adequate comparative or prospective evidence and without the parameters of the IDEAL recommendations. This means that uncertainties about the efficacy, effectiveness, and cost-effectiveness of RC remain, which has inherent risks for clinical practice. More rigorous methods for evaluation of surgical innovation are therefore recommended.

Two meta-analyses comparing RC and LC have been undertaken. The first (2016) included one RCT and 12 observational studies. The second (2017) included five RCTs (two of which were outside the inclusion criteria for our review) and 21 observational studies^11,12^. Neither identified any significant difference in complications, readmission rates, or hospital stay, although operating time and the incidence of postoperative incisional hernia were higher after RC^11^; however, these meta-analyses were based primarily on retrospective observational studies and therefore must be interpreted with caution due the presence of confounders, selection bias, and differences in study design^22^. Both studies highlighted the issue of heterogeneous outcomes, which reduced the number of studies available for meta-analysis. This finding is consistent with our own study and illustrates how heterogeneous outcomes can impair evidence synthesis^49–51^. The COMET (Core Outcome Measures in Effectiveness Trials) Initiative^52^ recommends the development of core outcome sets (an agreed minimum set of outcomes that should be measured and reported in all clinical trials of a specific disease or trial population)^53^ with an expectation that core outcomes will be collected and reported, making it easier for the results of studies to be compared, contrasted, and combined as appropriate^52,54^. Core outcome sets are increasingly mandated by journals before publication; streamlining the outcomes reported in robotic surgery would enable the efficacy and effectiveness of robotic procedures to be clearly detailed, subsequently optimizing transparency, maximizing patient benefit, and reducing harms.

To our knowledge, this review represents the first in-depth case study to summarize published evidence of how a robot-assisted procedure was adopted into clinical practice. Although the inclusion of all study types allowed a comprehensive review of the evidence base for RC, this study has some limitations. First, the exclusion of non-English language papers may have resulted in some relevant papers being missed. Second, reporting standards and expectations change with time; 19 of the included studies were published before the introduction of the IDEAL framework in 2009 and benchmarking such studies against these criteria may be considered unfair, although the principles underpinning IDEAL represent the foundations of evidence-based surgery. A third limitation is that the IDEAL Collaboration’s flow chart for determining stage of innovation was challenging to use because most papers did not provide information about technique descriptions or modifications, creating difficulties in distinguishing between 2a and 2b studies. Furthermore, many of the studies were difficult to classify given their retrospective nature; however, aside from the temporality of the study, other criteria to classify the IDEAL stage were met and they were therefore assigned stages while acknowledging this limitation. Retrospective categorization of studies to IDEAL stages has been recorded in the literature in line with this^55^. It is widely recognized that there is still a need for the quality of surgical research to improve, including the heavy reliance on retrospective study designs due to their inherent limitations.

In conclusion, this review highlights a lack of standardized reporting and adherence to IDEAL guidelines across studies underpinning the adoption of RC. This impairs surgeons’ ability to draw meaningful conclusions from available evidence and undertake shared decision-making with patients. Inadequate descriptions of inclusion criteria and heterogeneous outcome selection obstruct effective evidence synthesis and may cause research waste. Improved reporting would enable greater transparency and interpretability, and facilitate the safe, evidence-based adoption of new procedures into clinical practice. For RC, high-quality RCTs assessing patient-centred, surgeon-focused, and health economic outcomes are now required to guide its future use. We support greater adoption of tools to facilitate the generation of robust evidence for robotic surgical procedures, including the IDEAL reporting guidelines^56^ and robot-specific core outcome sets.

## Collaborators

**RoboSurg Collaborative**

A Vallance, A Wilkinson, A Smith, A Torkington, A Jones, A Abbas, BG Main, B Zucker, B Turner, CS Jones, C Thomas, C Hoffmann, DL Scroggie, D Henshall, EN Kirkham, E Boden, E Gull, E Sewart, F Dewi, F Wood, F Loro, F Hollowood, G Fowler, G Higginbotham, G Sellers, H Robertson, H Richards, I Hughes, I Handa, JM Blazeby, J Olivier, J Ramirez, J Rees, K Chalmers, K Siang Lee, L Dixon, L Leandro, L Paynter, L Huppler, L Gourbault, M Huttman, M Wijeyaratne, M Dewhurst, M Shah, M Kiandee, M Dada, NS Blencowe, O Brewster, P Lok, R Winayak, R Ranat, R Macefield, R Purves, R Lawrence, R Millar, S Biggs, S Lawday, S Dalmia, S Cousins, S Pathak, S Rozwadowski, T Robinson, T Perra, T Wei Leow, T Brankin-Frisby, W Baker, W Hurst, Y Embury Young.

## Supplementary Material

zrac116_Supplementary_Data

## Data Availability

We are willing to make our data, analytic methods, and study materials available to other researchers on request.
