# Erratum to: Quality of reporting of robot-assisted cholecystectomy in relation to the IDEAL recommendations: systematic review

**DOI:** 10.1093/bjsopen/zrad019

**Published:** 2023-03-07

This is an erratum to: Quality of reporting of robot-assisted cholecystectomy in relation to the IDEAL recommendations: systematic review, *BJS Open*, Volume 6, Issue 5, October 2022, zrac116, https://doi.org/10.1093/bjsopen/zrac116

The following changes have been made to the article. The title of the paper now reads: “Quality of reporting of robot-assisted cholecystectomy in relation to the IDEAL recommendations: systematic review” instead of “A systematic review of robot-assisted cholecystectomy to examine the quality of reporting in relation to the IDEAL recommendations: systematic review”.

In the original publication, there was an error in the first name of the corresponding author. This should read: “Samir Pathak” instead of “Sami Pathak”.

These emendations are now made in the article.

