# Supplementary material for: Quality of reporting of robot-assisted cholecystectomy in relation to the IDEAL recommendations: systematic review
Source: BJS Open. 2022 Oct 25;6(5):zrac116. doi: 10.1093/bjsopen/zrac116 (PMC9593068; doi:10.1093/bjsopen/zrac116)
Supplement: zrac116_Supplementary_Data [file zrac116_supplementary_data.docx]

**Collaborators**

**RoboSurg Collaborative:**

**Study management group (alphabetical):**

Biggs S, Blencowe NS, Dewi F, Dixon L, Huttman M, Jones CS, Kirkham EN, Main BG, Pathak S, Ramirez J, Robertson H, Scroggie DL, Zucker B

**Study design:**

Blazeby JM, Blencowe NS, Main BG, Pathak S

**Article identification:**

Biggs S, Blencowe NS, Dixon L, Hollowood F, Huttman M, Jones CS, Kirkham EN, Pathak S, Ramirez J, Rees J, Robertson H, Scroggie DL, Embury Young Y, Zucker B

**Data extraction and verification**:

Vallance A, Wilkinson A, Smith A, Torkington A, Jones A, Abbas A, Zucker B, Turner B, Thomas C, Hoffman C, Jones CS, Scroggie DL, Henshall D, Boden E, Kirkham EN, Gull E, Sewart E, Wood F, Dewi F, Loro F, Hollowood F, Fowler G, Higginbotham G, Sellers G, Robertson H, Richards H, Hughes I, Handa I, Olivier J, Rees J, Ramirez J, Siang Lee K, Dixon L, Leandro L, Paynter L, Huppler L, Gourbault L, Wijeyaratne M, Huttman M, Dewhurst M, Shah M, Kiandee M, Dada M, Blencowe NS, Brewster O, Lok P, Winayak R, Ranat R, Macefield R, Purves R, Lawrence R, Millar R, Lawday S, Pathak S, Dalmia S, Biggs S, Cousins S, Rozwadowski S, Robinson T, Perra T, Wei Leow T, Brankin-Frisby T, Baker W, Hurst W, Embury Young Y.

**Table S1: Preferred Reporting Items for Systematic Reviews and Meta-Analyses (PRISMA) checklist^17^**

| **Section and Topic** | **Item #** | **Checklist item** | **Location where item is reported** |
| --- | --- | --- | --- |
| **TITLE** | | |  |
| Title | 1 | Identify the report as a systematic review. | Title |
| **ABSTRACT** | | |  |
| Abstract | 2 | See the PRISMA 2020 for Abstracts checklist. | Sup Table 6 |
| **INTRODUCTION** | | |  |
| Rationale | 3 | Describe the rationale for the review in the context of existing knowledge. | Pages 3-4 |
| Objectives | 4 | Provide an explicit statement of the objective(s) or question(s) the review addresses. | Page 4 |
| **METHODS** | | |  |
| Eligibility criteria | 5 | Specify the inclusion and exclusion criteria for the review and how studies were grouped for the syntheses. | Pages 4-5 |
| Information sources | 6 | Specify all databases, registers, websites, organisations, reference lists and other sources searched or consulted to identify studies. Specify the date when each source was last searched or consulted. | Page 4 |
| Search strategy | 7 | Present the full search strategies for all databases, registers and websites, including any filters and limits used. | Sup Table 1 |
| Selection process | 8 | Specify the methods used to decide whether a study met the inclusion criteria of the review, including how many reviewers screened each record and each report retrieved, whether they worked independently, and if applicable, details of automation tools used in the process. | Page 5 |
| Data collection process | 9 | Specify the methods used to collect data from reports, including how many reviewers collected data from each report, whether they worked independently, any processes for obtaining or confirming data from study investigators, and if applicable, details of automation tools used in the process. | Page 5 |
| Data items | 10a | List and define all outcomes for which data were sought. Specify whether all results that were compatible with each outcome domain in each study were sought (e.g. for all measures, time points, analyses), and if not, the methods used to decide which results to collect. | Pages 5-7 |
|  | 10b | List and define all other variables for which data were sought (e.g. participant and intervention characteristics, funding sources). Describe any assumptions made about any missing or unclear information. | Pages 5-7 |
| Study risk of bias assessment | 11 | Specify the methods used to assess risk of bias in the included studies, including details of the tool(s) used, how many reviewers assessed each study and whether they worked independently, and if applicable, details of automation tools used in the process. | Page 6 |
| Effect measures | 12 | Specify for each outcome the effect measure(s) (e.g. risk ratio, mean difference) used in the synthesis or presentation of results. | Page 7 |
| Synthesis methods | 13a | Describe the processes used to decide which studies were eligible for each synthesis (e.g. tabulating the study intervention characteristics and comparing against the planned groups for each synthesis (item #5)). | Page 7 |
|  | 13b | Describe any methods required to prepare the data for presentation or synthesis, such as handling of missing summary statistics, or data conversions. | NA* |
|  | 13c | Describe any methods used to tabulate or visually display results of individual studies and syntheses. | NA* |
|  | 13d | Describe any methods used to synthesize results and provide a rationale for the choice(s). If meta-analysis was performed, describe the model(s), method(s) to identify the presence and extent of statistical heterogeneity, and software package(s) used. | Page 7 |
|  | 13e | Describe any methods used to explore possible causes of heterogeneity among study results (e.g. subgroup analysis, meta-regression). | NA* |
|  | 13f | Describe any sensitivity analyses conducted to assess robustness of the synthesized results. | NA* |
| Reporting bias assessment | 14 | Describe any methods used to assess risk of bias due to missing results in a synthesis (arising from reporting biases). | NA* |
| Certainty assessment | 15 | Describe any methods used to assess certainty (or confidence) in the body of evidence for an outcome. | NA* |
| **RESULTS** | | |  |
| Study selection | 16a | Describe the results of the search and selection process, from the number of records identified in the search to the number of studies included in the review, ideally using a flow diagram. | Figure 1 |
|  | 16b | Cite studies that might appear to meet the inclusion criteria, but which were excluded, and explain why they were excluded. | Figure 1 |
| Study characteristics | 17 | Cite each included study and present its characteristics. | Table 2 |
| Risk of bias in studies | 18 | Present assessments of risk of bias for each included study. | Sup Table 3 |
| Results of individual studies | 19 | For all outcomes, present, for each study: (a) summary statistics for each group (where appropriate) and (b) an effect estimate and its precision (e.g. confidence/credible interval), ideally using structured tables or plots. | NA* |
| Results of syntheses | 20a | For each synthesis, briefly summarise the characteristics and risk of bias among contributing studies. | Pages 7-9 |
|  | 20b | Present results of all statistical syntheses conducted. If meta-analysis was done, present for each the summary estimate and its precision (e.g. confidence/credible interval) and measures of statistical heterogeneity. If comparing groups, describe the direction of the effect. | NA* |
|  | 20c | Present results of all investigations of possible causes of heterogeneity among study results. | NA* |
|  | 20d | Present results of all sensitivity analyses conducted to assess the robustness of the synthesized results. | NA* |
| Reporting biases | 21 | Present assessments of risk of bias due to missing results (arising from reporting biases) for each synthesis assessed. | NA* |
| Certainty of evidence | 22 | Present assessments of certainty (or confidence) in the body of evidence for each outcome assessed. | NA* |
| **DISCUSSION** | | |  |
| Discussion | 23a | Provide a general interpretation of the results in the context of other evidence. | Pages 9-10 |
|  | 23b | Discuss any limitations of the evidence included in the review. | Pages 10-11 |
|  | 23c | Discuss any limitations of the review processes used. | Pages 10-11 |
|  | 23d | Discuss implications of the results for practice, policy, and future research. | Page 11 |
| **OTHER INFORMATION** | | |  |
| Registration and protocol | 24a | Provide registration information for the review, including register name and registration number, or state that the review was not registered. | Title page |
|  | 24b | Indicate where the review protocol can be accessed, or state that a protocol was not prepared. | Page 4 |
|  | 24c | Describe and explain any amendments to information provided at registration or in the protocol. | NA |
| Support | 25 | Describe sources of financial or non-financial support for the review, and the role of the funders or sponsors in the review. | Title page |
| Competing interests | 26 | Declare any competing interests of review authors. | Title page |
| Availability of data, code and other materials | 27 | Report which of the following are publicly available and where they can be found: template data collection forms; data extracted from included studies; data used for all analyses; analytic code; any other materials used in the review. | On request |

*****The current review is presented as a narrative synthesis of all studies reporting outcomes of robot-assisted cholecystectomy. Some points are therefore, not applicable.

**Table S2: Preferred Reporting Items for Systematic Reviews and Meta-Analyses (PRISMA) for abstracts checklist^17^.**

| **Section and Topic** | **Item #** | **Checklist item** | **Reported (Yes/No)** |
| --- | --- | --- | --- |
| **TITLE** | | |  |
| Title | 1 | Identify the report as a systematic review. | Yes |
| **BACKGROUND** | | |  |
| Objectives | 2 | Provide an explicit statement of the main objective(s) or question(s) the review addresses. | Yes |
| **METHODS** | | |  |
| Eligibility criteria | 3 | Specify the inclusion and exclusion criteria for the review. | Yes |
| Information sources | 4 | Specify the information sources (e.g. databases, registers) used to identify studies and the date when each was last searched. | Yes |
| Risk of bias | 5 | Specify the methods used to assess risk of bias in the included studies. | No |
| Synthesis of results | 6 | Specify the methods used to present and synthesise results. | Yes |
| **RESULTS** | | |  |
| Included studies | 7 | Give the total number of included studies and participants and summarise relevant characteristics of studies. | Yes |
| Synthesis of results | 8 | Present results for main outcomes, preferably indicating the number of included studies and participants for each. If meta-analysis was done, report the summary estimate and confidence/credible interval. If comparing groups, indicate the direction of the effect (i.e. which group is favoured). | Yes |
| **DISCUSSION** | | |  |
| Limitations of evidence | 9 | Provide a brief summary of the limitations of the evidence included in the review (e.g. study risk of bias, inconsistency and imprecision). | No* |
| Interpretation | 10 | Provide a general interpretation of the results and important implications. | Yes |
| **OTHER** | | |  |
| Funding | 11 | Specify the primary source of funding for the review. | Title page |
| Registration | 12 | Provide the register name and registration number. | Title page |

*Given the nature of this study, “limitations of evidence” is less relevant given that the study itself assessed the limitations of evidence.

**Table S3: MEDLINE search strategy**

| 1 | robotics/ (58241) |
| --- | --- |
| 2 | robot assisted surgery/ (9669) |
| 3 | robotic surgical procedure/ or robotic surgical procedures/ (10497) |
| 4 | computer assisted surgery/ or surgery, computer-assisted/ (26352) |
| 5 | computer assisted surgery system/ (120) |
| 6 | robotic surgical device/ or robotic console/ or robotic navigation system/ or robotic surgical system/ or robotic catheter system/ or robotic neurological surgical equipment/ or robotic orthopedic surgical system/ or robotic hip surgery system/ or robotic knee surgery system/ or robotic spine surgical system/ (2000) |
| 7 | robotic surgical instrument/ or robotic endoscope/ or robotic needle driver/ or robotic retractor/ or robotic scissors/ or robotic forceps/ or robotic dissecting forceps/ or robotic grasping forceps/ or robotic sealing device/ or robotic stapler/ or robotic vessel sealer/ (225) |
| 8 | robot assisted.hw. (12430) |
| 9 | (robot* or telerobot* or tele-robot* or telesurger* or tele-surger*).ti,ab,kw. (110930) |
| 10 | (computer assist* adj5 (operation* or procedur* or surger* or surgeon? or surgical*)).ti,ab,kw. (5791) |
| 11 | (da Vinci? or davinci?).mp. (11707) |
| 12 | (remote adj2 (operation* or procedur* or surger* or surgeon? or surgical*)).ti,ab,kw. (1478) |
| 13 | ("state of the art" adj (surger* or surgical*)).ti,ab,kw. (213) |
| 14 | or/1-13 (151495) |
| 15 | (Arthrobot* or Aesop* or Hugo* or Mako* or MiroSurge* or Monarch* or Neuroarm* or Probot* or Revo-I or ROBODOC).mp. (7829) |
| 16 | (Senhance* or Sina or Sofie or SPIDER or SPORT or SurgiBot or Telelap or Unimation or Puma or Versius or ZEUS).mp. (167585) |
| 17 | 15 or 16 (175202) |
| 18 | *biliary tract surgery/ or cholecystectomy/ or laparoscopic cholecystectomy/ (78690) |
| 19 | *biliary tract surgical procedures/ or cholecystectomy/ or cholecystectomy, laparoscopic/ (81107) |
| 20 | (cholecystecto* or colecystecto*).mp. (92356) |
| 21 | or/18-20 (97054) |
| 22 | 14 and 21 (1663) |
| 23 | 17 and 21 (195) |
| 24 | 22 or 23 (1721) |
| 25 | (conference abstract or conference review).pt. (3738512) |
| 26 | 24 not 25 (1358) |
| 27 | remove duplicates from 26 (906) |

**Table S4: Outcome domains and definitions**

| **Domain** | **Definition** | **Examples** |
| --- | --- | --- |
| Complications | An untoward medical occurrence as a result of the operation at any time from surgery to last follow up^58^ | Readmission, bile leak, pneumonia. |
| Technical and operative | Outcomes relating to the robot and/or operation itself. | Operative time, docking time, estimated blood loss. |
| Survival | Death of a patient at any time during the study period. | 30-day mortality |
| Laboratory and imaging | Measurable observations relating to a patient’s underlying physiology or anatomy, including blood tests and imaging. | Blood tests, cholangiogram findings |
| Patient centred | Including patient reported outcomes, functional outcomes and symptoms which come directly from the patient, without interpretation of the patient’s response by a clinician or anyone else^59^ | Quality of life, post-operative pain level, time to oral diet |
| Trends and learning curve | Changes to clinical practice with time and/or the rate of acquisition of surgical skills as defined by the study authors. | Change in operation time |
| Health economic and resource use | Outcomes relating to direct and/or indirect costs of surgery or the use of health services, including length of stay | Length of stay, financial cost of procedure |
| Surgeon experience | Outcomes relating to a surgeon’s subjective experience of the technology and/or operation | Surgeon satisfaction |

**Table S5. Details about the RCTs and prospective comparative studies (n=5)**

| **Author, year** | **Study design** | **Number of patients** | **Comp** | **Differences in baseline characteristics** | | | | | | | | | | | | |
| --- | --- | --- | --- | --- | --- | --- | --- | --- | --- | --- | --- | --- | --- | --- | --- | --- |
|  |  |  |  | **Age** | | **Sex (% male)** | | **ASA** | | **BMI** | | **Indication** | | **Other characteristics reported** | **Were cohorts matched at baseline?** |  |
|  |  |  |  | **RC** | **Comp** | **RC** | **Comp** | **RC** | **Comp** | **RC** | **Comp** | **RC** | **Comp** |  |  |  |
| Kudsi 2017^24^ | RCT | 136 (83 RC, 53 LC) | LC | 46.8 | 46.5 | 21 | 7 | ASA 1 20% | ASA 1 21% | 30.4 | 31.7 | CC 30.1% | CC 26.4% | Ethnicity, comorbidities | Significantly more females in LC group |  |
| Pietrabissa 2016^23^ | RCT | 60 (30 in each arm) | LC | NR | NR | NR | NR | NR | NR | NR | NR | NR | NR | NR | “Groups were comparable in each arm” |  |
| Heemskerk, 2014^22^ | RCT | 22 (11 in each arm) | LC | NR | NR | NR | NR | NR | NR | NR | NR | NR | NR | NR | NR |  |
| Kane 2019^60^ | Prospective cohort | 1166 (106 RC, 1060 SILC) | SILC | 41.5 | 43 | 28.3 | 29.9 | NR | NR | 30.1 | 30.2 | NR | NR | Comorbidities, smoking status | Propensity score matching |  |
| Buchs 2013^61^ | Prospective cohort | 44 (23 RC, 21 RC + C) | RC + C | 47.3 | 48 | 47.8 | 9.5 | Mean 1.7 | Mean 1.8 | 27 | 27 | NR | NR | NR | More men in RC group |  |

RC = robotic cholecystectomy, SILC = single incision laparoscopic cholecystectomy, ASA = American Society Anaesthesiologists score, Comp = comparator, NR = not reported, CC = chronic cholecystitis, RC + C = RC with cholangiography
